# Polycyclic Aromatic Hydrocarbons in Residential Dust: Sources of Variability

**DOI:** 10.1289/ehp.1205821

**Published:** 2013-03-05

**Authors:** Todd P. Whitehead, Catherine Metayer, Myrto Petreas, Monique Does, Patricia A. Buffler, Stephen M. Rappaport

**Affiliations:** 1 School of Public Health, University of California, Berkeley, Berkeley, California, USA; 2 California Department of Toxic Substances Control, Environmental Chemistry Laboratory, Berkeley, California, USA

**Keywords:** environmental exposures, house dust, polycyclic aromatic hydrocarbons

## Abstract

Background: There is interest in using residential dust to estimate human exposure to environmental contaminants.

Objectives: We aimed to characterize the sources of variability for polycyclic aromatic hydrocarbons (PAHs) in residential dust and provide guidance for investigators who plan to use residential dust to assess exposure to PAHs.

Methods: We collected repeat dust samples from 293 households in the Northern California Childhood Leukemia Study during two sampling rounds (from 2001 through 2007 and during 2010) using household vacuum cleaners, and measured 12 PAHs using gas chromatography–mass spectrometry. We used a random- and a mixed-effects model for each PAH to apportion observed variance into four components and to identify sources of variability.

Results: Median concentrations for individual PAHs ranged from 10 to 190 ng/g of dust. For each PAH, total variance was apportioned into regional variability (1–9%), intraregional between-household variability (24–48%), within-household variability over time (41–57%), and within-sample analytical variability (2–33%). Regional differences in PAH dust levels were associated with estimated ambient air concentrations of PAH. Intraregional differences between households were associated with the residential construction date and the smoking habits of residents. For some PAHs, a decreasing time trend explained a modest fraction of the within-household variability; however, most of the within-household variability was unaccounted for by our mixed-effects models. Within-household differences between sampling rounds were largest when the interval between dust sample collections was at least 6 years in duration.

Conclusions: Our findings indicate that it may be feasible to use residential dust for retrospective assessment of PAH exposures in studies of health effects.

Semivolatile chemicals accumulate on dust particles, and dust that is trapped deep within a carpet can be a permanent reservoir for these chemicals (Roberts et al. 2009). Thus, polycyclic aromatic hydrocarbon (PAH) concentrations in carpet dust should reflect long-term average levels of residential PAH contamination.

Although many researchers have measured PAHs in dust, few have characterized the variability of dust measurements within and between households over time (Egeghy et al. 2005; Whitehead et al. 2012). When estimating the health effects related to a chemical exposure, the variance ratio (i.e., ratio of within-subject variability to between-subject variability) is predictive of the underestimation of the relationship between an exposure and a health effect (Armstrong 1998). We have found that variance ratios for PAHs in residential dust are generally modest when repeat dust samples are collected at semiannual intervals (Whitehead et al. 2012). Thus, one dust measurement should be sufficient to characterize the average levels of contamination found in a residence over a year or so. However, the magnitude of temporal variability that exists in residential-dust measurements over years or decades has not been estimated and may be important for accurately assessing long-term exposure.

Our objective in this investigation was to characterize the long-term variability of PAH concentrations in residential dust. We analyzed 12 PAHs in repeated residential-dust samples collected at intervals of 3–8 years. Because long-term exposures to PAHs have been associated with adverse health effects (Boffetta et al. 1997), and dust ingestion and inhalation can be significant sources of PAH exposures (Chuang et al. 1999), we also identified predictors of residential-dust PAH levels.

## Methods

*Study population.* The Northern California Childhood Leukemia Study is a case–control study of childhood leukemia conducted in the San Francisco Bay area and California Central Valley that seeks to identify genetic and environmental risk factors for childhood leukemia. Cases 0–14 years of age were ascertained from pediatric clinical centers; controls, matched to cases on date of birth, sex, race, and Hispanic ethnicity, were selected from the California birth registry (California Department of Public Health, Sacramento, CA). Residential dust samples were collected from study homes as one strategy for assessing relevant environmental exposures. Case and control participants who were enrolled in the study from December 1999 through November 2007 were eligible for initial residential-dust collection if they were 0–7 years old and lived in the same home they had occupied at the time of diagnosis (or a similar reference date for controls). Subsequently, in 2010, participants in the initial dust collection were eligible for a second dust collection if they were still living in the same home. Among 629 participants in the initial dust collection, 225 were eligible for a second dust collection and 204 participated in the second dust collection. We successfully analyzed two dust samples for PAHs in 201 homes and successfully analyzed only the second dust sample for PAHs in three homes. For an additional 89 participants in the initial dust collection who were ineligible for the second dust collection, we also analyzed one dust sample for PAHs, as described below. We obtained written informed consent from the children’s parents and study protocols were approved by the institutional review board at the University of California, Berkeley.

*Collection of residential dust*. During the first round of dust sampling (2001–2007), we collected vacuum cleaner dust and administered a questionnaire during an in-home visit. During the second round of dust sampling (2010), we interviewed participants via telephone and instructed them to mail their vacuum cleaner bags (or the contents of their vacuum cleaner canisters) to the study center in prepaid parcels. The median interval between repeated sample collections was 4.8 years (range, 2.6–8.6 years). We stored dust samples away from heat (≤ 4^o^C) and light before chemical analysis. We previously analyzed the dust samples from the first round of dust collection for nine PAHs (Whitehead et al. 2009); however, for consistency, the dust samples from the first round of dust collection were reextracted and reanalyzed alongside the samples from the second round of dust collection according to the protocol described below.

*Laboratory analysis of PAHs*. We homogenized and fractionated the dust samples using a mechanical sieve shaker equipped with a 100-mesh sieve to obtain dust particles < 150 μm. Portions of fine dust (0.2 g) were spiked with an internal standard (50 ng of d_12_-benzo[*a*]pyrene), extracted via accelerated solvent extraction, purified by silica-gel column chromatography and gel permeation chromatography, concentrated to 250 μL, solvent exchanged into tetradecane, and spiked with a recovery standard (50 ng of d_10_-pyrene). Finally we analyzed 12 PAHs (phenanthrene, anthracene, fluoranthene, pyrene, benzo[*a*]anthracene, chrysene, benzo[*b*]fluoranthene, benzo[*k*]fluoranthene, benzo[*a*]pyrene, indeno[*1,2,3-c,d*]pyrene, dibenzo[*a,h*]anthracene, and benzo[*g,h,i*]perylene) using gas chromatography–mass spectrometry in the multiple ion detection mode. The chromatographic separation used a DB-5 column (60 m, 0.25 mm i.d., 0.25 μm film) that was programmed from 150^o^C to 250^o^C at 25^o^C per minute, and then from 250^o^C to 315^o^C at 2.5^o^C per minute. We analyzed a six-point calibration curve (range, 20–62,500 ng/mL) at the beginning and the end of sample analysis and a single point standard with each sample set. The analytical protocol was validated using replicate dust samples of National Institute of Standards and Technology (NIST) Standard Reference Material (SRM) 2585 (NIST, Gaithersburg, MD). For all validation replicates, measured concentrations of each PAH were generally within 30% of the NIST certified value (maximum error of 55%), and the sum of the PAHs was within 5% of the sum of the NIST certified values.

*Quality control samples*. We analyzed samples in batches of 12, with each batch consisting of 8 samples, 1 method blank, 1 duplicate sample pair (i.e., two 200-mg portions of fine dust taken from the same vacuum cleaner), and 1 interbatch quality control sample (i.e., a 200-mg portion of fine dust taken from the quality-control vacuum cleaner). Because we prepared and analyzed an interbatch quality control replicate alongside each successive sample batch, the interbatch quality control results illustrate the reproducibility of the dust preparation and analytical methods over the course of the study. Likewise, the duplicate samples illustrate the reproducibility of the dust preparation and analytical methods within each sample batch. For some batches, we replaced the interbatch quality control sample with the SRM 2585 dust sample. The SRM 2585 dust was vigorously homogenized, so results obtained from any 200-mg replicate should be highly reproducible. To demonstrate the optimal reproducibility of our method, we analyzed three pairs of duplicate SRM 2585 dust samples concurrently. To compare the magnitude of variability observed in the four types of quality control samples, we calculated the relative percent difference (RPD) between matched samples [for details regarding RPD calculations, see Supplemental Material, “Quality control samples” (http://dx.doi.org/10.1289/ehp.1205821)].

*Questionnaire responses*. Parents initially responded to structured in-home interviews designed to ascertain information relevant to childhood leukemia. Subsequently, households participating in the second dust collection (*n* = 204) completed an additional telephone questionnaire designed to ascertain information about sources of residential chemical exposures. The latter questionnaire covered topics related to sources of indoor PAHs, including cigarette smoking, appliances, cooking practices, and shoe removal habits, as well as residential characteristics such as residential construction date, type, and square footage [see Supplemental Material, “Questions used to create variables for mixed-effects models” (http://dx.doi.org/10.1289/ehp.1205821)].

**Figure 1:**
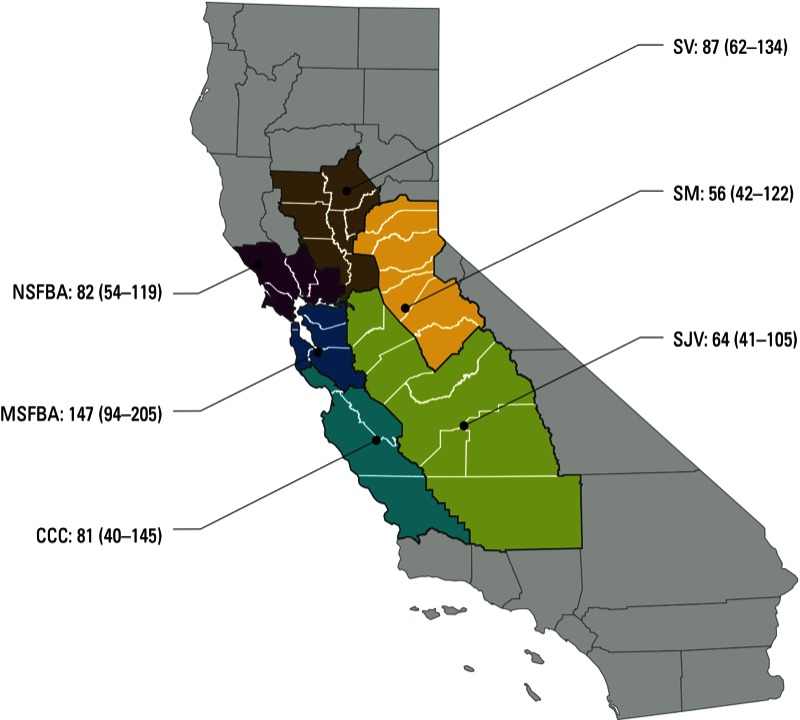
Regional variability in median (interquartile range) benzo[*g,h,i*]perylene concentrations (ng/g) in dust samples collected from 290 residences in the Northern California Childhood Leukemia Study, 2001–2007. Abbreviations: MSFBA, the metropolitan San Francisco Bay area (includes Alameda, Contra Costa, Santa Clara, San Francisco, and San Mateo counties); NSFBA, the northern San Francisco Bay area (includes Marin, Napa, Solano, and Sonoma counties); SV, the Sacramento Valley (includes Butte, Colusa, Glenn, Sacramento, Sutter, Yolo, and Yuba counties); SM, the Sierra Mountains (includes Amador, Calaveras, El Dorado, Mariposa, Nevada, Placer, and Tuolumne counties); SJV, the San Joaquin Valley (includes Fresno, Kern, Kings, Madera, Merced, San Joaquin, Stanislaus, Tulare counties); CCC, the California central coast (includes Monterey, San Benito, San Luis Obispo, and Santa Cruz counties).

*Geographic information*. We used a global positioning device to determine the latitude and longitude for each residence and classified each residence as belonging to one of six geographic regions (Figure 1). We estimated ambient air PAH concentrations at a census tract resolution using results from the U.S. Environmental Protection Agency (EPA) 2005 National-Scale Air Toxics Assessment (U.S. EPA 2011). The U.S. EPA assessment employed a National Emissions Inventory (U.S. EPA 2012) to estimate ambient air concentrations of 16 PAHs (including the 12 PAHs measured in this study, as well as acenaphthene, acenaphthylene, fluorine, and naphthalene) attributable to emissions from major stationary sources (e.g., power plants), area sources (e.g., commercial buildings), and mobile sources (e.g., automobiles). To distinguish between traffic emissions and emissions from other urban PAH sources, we considered ambient concentrations of PAH attributable to mobile sources and ambient concentrations of PAH attributable to area sources as two independent determinants of PAH levels in residential dust. Since the association between ambient PAH estimates and residential-dust PAH concentrations was nonlinear, we used the rank order of these census tract–level estimates for all regression analyses.

*Random-effects models*. To apportion the observed variance in PAH concentrations into four components describing regional variability, intraregional between-household variability, within-household variability over time, and within-sample analytical variability we used a hierarchical random-effects model,

*Y_hijk_ = ln*(*X_hijk_*) *=* µ*_Y_ + b_h_ + b_hi_ + b_hij_ + e_hijk_,* [1]

for *h* = 1,2,…,6 regions; *i* = 1,2,…,294 households (i.e., 293 study residences and the interbatch quality control residence); *j* = sampling round 1 or 2; and *k* = 1,2…,40 replicate samples from the same vacuum bag, where *X_hijk_* = the residential-dust PAH concentration for the *i*th household in the *h*th region, from the *k*th subsample of the *j*th repeated measurement; *Y_hijk_* = the natural log-transform of *X_hijk_*; μ*_Y_* = the true (logged) mean residential-dust PAH concentration for the population; *b_h_* = μ*_Yh_*–μ*_Y_*, and represents the random deviation of the *h*th region’s true mean (logged) residential-dust PAH concentration, μ*_Yh_*, from μ*_Y_*; *b_hi_* = μ*_Yhi_*–μ*_Yh_*, and represents the random deviation of the *i*th household’s true mean (logged) residential-dust PAH concentration, μ*_Yhi_*, from μ*_Yh_*; *b_hij_* = μ*_Yhij_*–μ*_Yhi_*, and represents the random deviation of the *j*th measurement’s true mean (logged) residential-dust PAH concentration, μ*_Yhij_*, from μ*_Yhi_*; *e_hijk_* = *Y_hijk_*–μ*_Yhij_*, and represents the random deviation of the observed (logged) residential-dust PAH concentration, *Y_hijk_*, from μ*_Yhij_* for the *i*th household in the *h*th region on the *j*th repeated measurement.

We assume *b_h_*, *b_hi_*, *b_hij_*, and *e_hijk_* are mutually independent and normally distributed random variables, with means of zero and variances of σ^2^*_BR_*, σ^2^*_BH_*, σ^2^*_WH_*, and σ^2^*_WS_*, representing the between-region variability, the intraregional between-household variability, the within-household variability over time, and the within-sample analytical variability, respectively. Using the spatial analyst function in ArcGIS (ESRI, Redlands, CA), we estimated Moran’s *I* statistic of spatial autocorrelation and confirmed that household-level random effects from model 1 were independent. Using PROC MIXED (version 9.1; SAS Institute Inc., Cary, NC), we fit the model described in Equation 1 and estimated variance components (σ^^2^^*_BR_*, σ^^2^^*_BH_*, σ^^2^^*_WH_*, σ^^2^^*_WS_*, σ^^2^^*_Total_* = σ^^2^^*_BR_* + σ^^2^^*_BH_* + σ^^2^^*_WH_ +* σ^^2^^*_WS_*) and variance ratios [λ = (σ^^2^^*_WH_* + σ^^2^^*_WS_*)/(σ^^2^^*_BR_* + σ^^2^^*_BH_*)]. As previously described (Whitehead et al. 2012), for each PAH, we used the magnitude of the variance ratio to estimate the potential impact of measurement error on an odds ratio (*OR*_True_ = 2.0) for a hypothetical case–control study that employs a single dust sample to assess exposure to PAHs (*OR*_Biased_ = exp [ln(*OR*_True_)/(1 + λ)].

To assess the impact of unequal within-household variance in case and control homes on variance ratio estimates, we used a second random-effects model (model 2) to apportion variance into three components for between-household variability (in all homes), within-household variability in case homes, and within-household variability in control homes, as described in detail in Supplemental Material, “Random-effects Model 2” (http://dx.doi.org/10.1289/ehp.1205821).

*Mixed-effects models*. Complete model specifications for the mixed-effects models are provided in Supplemental Material, pp. 3–6 (http://dx.doi.org/10.1289/ehp.1205821). In brief, we used mixed-effects models to identify sources of variability for each hierarchical level. In addition to the model 1 random effects, we included two fixed effects for neighborhood-level covariates in model 3: the rank order of estimated ambient concentrations of PAH attributable to emissions from area sources, and the rank order of estimated ambient concentrations of PAH attributable to emissions from mobile sources for the census tracts in the study. Likewise, in addition to the model 1 random effects, we included seven fixed effects for residential covariates in model 4: regular smoking inside or outside of the residence, residence construction date, residence is apartment or condominium, regular shoe removal by residents in home, < 25% of residence is carpeted, residence square footage is < 1,750 ft^2^, and residence has at least two forms of combustion-based heating (i.e., gas or kerosene heat, fireplace, wood-burning stove, or steam radiator). Similarly, in addition to the model 1 random effects, we included two fixed effects for temporal covariates in model 5: the date of dust collection and the sequence of the laboratory analysis. In the fully saturated model 6, we included the random effects from model 1 as well as neighborhood, residential, and temporal covariates from models 3–5.

We fit each of the above mixed-effects models (models 3–6) for 451 observations with covariate data (i.e., 405 samples collected from 204 homes during repeat sampling rounds and 46 duplicate samples) and excluded the 139 observations without covariate data (i.e., 40 interbatch quality control replicates and 89 samples with 10 duplicates collected during round 1). For comparison, we re-ran the random-effects model (model 1) using this set of 451 observations. In a stratified analysis we fit model 6 using the case and control data separately to evaluate whether the fixed-effects estimates differed by case–control status.

Time trends in PAH concentrations may have differed by region. Model 7 includes a unique fixed effect for the time trend in PAH for each region in addition to the random and fixed effects in model 6.

To evaluate the influence of the time interval between repeat dust collections on within-household variability, model 8 includes four random effects representing the between-household variability (in all homes) and the within-household variability for households with various time intervals between sample collections (i.e., < 4 years, 4–6 years, ≥ 6 years) in addition to the fixed effects used in model 6.

*Data imputation*. We determined method reporting limits (MRL) for each PAH on a batch-by-batch basis according to the contamination measured in the method blank (i.e., MRL = 3 × mass of PAH in the method blank). We replaced each value below the MRL (Table 1) with five imputations randomly selected from a log-normal distribution describing the PAH concentrations. The imputation procedure was restricted so that all replacement values were below the MRL. Additionally, some participants were unable or unwilling to complete all aspects of the questionnaires, and we also replaced missing covariate data using multiple imputation (e.g., for each of nine respondents who did not know their residence’s date of construction, we imputed five replacement dates). Ultimately, we created five complete data sets with a different imputed value for each missing value, performed regression analyses separately on each data set, and combined the results to produce confidence intervals that reflected the uncertainty created by the missing values. Moreover, we were unable to pinpoint six residences using the global positioning system, so we approximated their location using postal codes and replaced each missing census tract–level ambient PAH estimate with the corresponding county-level ambient PAH estimate.

**Table 1:** Summary statistics for PAH measurements in dust collected from 293 residences in the Northern California Childhood Leukemia Study.

PAH	Molecular weight	MRLa	Dust collection round 1, 2001–2007 (n = 290)		Dust collection round 2, 2010 (n = 204)		rS between roundsc
			> MRL [n (%)]	Median concentrationb (ng/g)	> MRL [n (%)]	Median concentrationb (ng/g)	
Phenanthrene	178	99.0	125 (43)	120	140 (69)	100	0.34
Anthracene	178	6.1	211 (73)	14	190 (93)	14	0.30
Fluoranthene	202	38.0	247 (85)	130	196 (96)	130	0.42
Pyrene	202	28.0	279 (96)	160	203 (100)	190	0.39
Benzo[a]anthracene	228	1.1	288 (99)	31	204 (100)	29	0.45
Chrysene	228	10.0	286 (99)	100	204 (100)	110	0.50
Benzo[b]fluoranthene	252	4.3	285 (98)	71	203 (100)	61	0.52
Benzo[k]fluoranthene	252	3.2	285 (98)	44	203 (100)	41	0.49
Benzo[a]pyrene	252	3.8	285 (98)	43	204 (100)	34	0.47
Indeno[1,2,3-c,d]pyrene	276	1.4	286 (99)	47	202 (99)	35	0.54
Dibenzo[a,h]anthracene	278	0.8	285 (98)	14	204 (100)	10	0.44
Benzo[g,h,i]perylene	276	4.3	288 (99)	100	204 (100)	86	0.57
PAHs are ordered by molecular weight from lightest to heaviest. aMedian method reporting limit from 494 samples. bMedian was estimated using imputed values for observations below the method reporting limit. cEach rS value was significantly greater than 0 (p < 0.001).							

To evaluate the impact of the multiple imputation procedure on estimates of variance components and fixed effects, for each PAH we fit model 1 using only the observations above the limit of detection; also, we fit model 6 using *a*) only the observations above the limit of detection, *b*) only the observations with complete covariate data, and *c*) only the observations above the limit of detection with complete covariate data. For most PAHs, the estimated variance components were similar in the limited and full analyses; however, for phenanthrene (and to a lesser extent anthracene and fluoranthene), the within-sample analytical variability was smaller in the limited analysis (data not shown). The fixed effects produced in the limited and full analyses were qualitatively similar [i.e., each fixed effect that was significant (*p*-value < 0.05) in the full analyses retained the same direction in the limited analyses with only minor changes in magnitude and significance observed (data not shown)].

## Results

Table 1 shows summary statistics for PAH measurements made in 293 California households. We detected individual PAHs in 43–99% of dust samples collected in round 1 and in 69–100% of dust samples collected in round 2. Median PAH concentrations ranged from 14 to 16 ng/g for dust samples collected in round 1 and from 10 to 190 ng/g for dust samples collected in round 2. Spearman rank correlation coefficients (*r*_S_) for interround comparisons of dust concentrations of PAHs ranged from 0.30 to 0.57 (*p* < 0.001 for all PAHs).

*Variability in quality control samples*. Table 2 shows the average RPDs between concentrations of PAHs in matched pairs of various quality control dust samples. For three pairs of SRM 2585 dust samples, the average RPD between PAH concentrations in duplicate samples analyzed on the same day was generally modest (average RPD range, 1.2–15%, 11 of 12 PAHs with RPD < 5%; Table 2). In contrast, when 17 replicate SRM 2585 dust samples were analyzed over the course of the study (interbatch), the average RPDs between randomly selected pairs of PAH concentrations were generally larger (average RPD range, 3.1–28%; Table 2). In particular, concentrations of the lower-molecular-weight PAHs measured in the SRM 2585 dust samples were less reproducible when analyzed repeatedly over a long period of time. Compared with the average RPDs for pairs of duplicate SRM 2585 dust samples (Table 2), the average RPDs between concentrations of PAHs measured in pairs of duplicate samples were relatively large (average RPD range, 5.6–17%; Table 2). The RPD between a randomly selected pair of PAH concentrations was generally greatest for the 40 replicate quality control samples analyzed alongside successive sample batches over the course of the study (average RPD range, 10–33%; Table 2).

**Table 2:** Average relative percent difference between concentrations of PAHs in matched quality control (QC) samples. PAHs are ordered by molecular weight from lightest to heaviest.

PAH	Intrabatch QC, SRM 2585a	Interbatch QC, SRM 2585b	Intrabatch QC, duplicate samplesc	Interbatch QC, replicate samplesd
Phenanthrene	1.5	21.0	8.9	33
Anthracenee		28.0	17.0	26
Fluoranthene	3.2	25.0	10.0	23
Pyrene	3.8	24.0	5.6	17
Benzo[a]anthracene	1.2	9.3	13.0	18
Chrysene	1.2	15.0	8.0	10
Benzo[b]fluoranthene	15.0	8.4	11.0	15
Benzo[k]fluoranthene	3.9	4.5	13.0	19
Benzo[a]pyrene	1.8	3.1	14.0	25
Indeno[1,2,3-c,d]pyrene	1.7	7.7	10.0	19
Dibenzo[a,h]anthracene	1.5	12.0	14.0	20
Benzo[g,h,i]perylene	1.6	12.0	6.2	15
aThree pairs of duplicate SRM 2585 dust samples analyzed concurrently. bSeventeen replicate SRM 2585 dust samples analyzed over the course of the study. cFifty-six pairs of duplicate samples, each pair analyzed concurrently. dForty replicate quality control samples analyzed over the course of the study. eIntrabatch quality control SRM 2585 samples were not analyzed for anthracene because an appropriate calibration standard was not available.				

*Random-effects modeling*. Table 3 shows estimated variance components for each PAH from the hierarchical random-effects model (model 1) with corresponding variance ratios. Between-region variability accounted for 0.8–9.2% of the total variability in residential-dust PAH measurements and regional variability was greater for heavier PAHs (e.g., benzo[*g,h,i*]perylene) compared with lighter PAHs (e.g., phenanthrene). For example, Figure 1 shows the regional variability of benzo[*g,h,i*]perylene concentrations. Intraregional between-household variability accounted for 24–48% of the total variability in residential-dust PAH measurements, and between-household variability was greater for heavier PAHs (e.g., benzo[*k*]fluoranthene) compared with lighter PAHs (e.g., phenanthrene). Within-household variability over time accounted for 41–57% of the total variability in residential-dust PAH measurements, and there was no apparent pattern related to PAH molecular weight. Within-sample analytical variability accounted for 2.1–33% of the total variability in residential-dust PAH measurements, and analytical variability was greater for lighter PAHs (e.g., phenanthrene). The variance ratio (i.e., the ratio of the sum of the within-household variability over time and the within-sample analytical variability to the sum of the regional variability and the intraregional between-household variability) ranged from 0.9 to 3.0. Based on these variance ratios, we would expect ORs to be attenuated from a true value of 2.0 to as low as 1.2 (range, 1.2–1.4) in a hypothetical case–control study that employs a single dust sample to assess exposure to PAHs.

**Table 3:** Estimated variance components from the random-effects model (model 1*^a^*) with corresponding variance ratios.

PAH	Variance component estimate (95% CI)				Variance ratiob	ORBiasedc	Percent of total variance			
	Between- region	Intraregional between-household	Within- household over time	Within- sample analytical			Between-region	Intraregional between-household	Within-household over time	Within-sample analytical
Phenanthrene	0.004 (–0.01, 0.02)	0.14 (0.05, 0.23)	0.23 (0.12, 0.35)	0.19 (0.10, 0.27)	3.0	1.2	0.8	24	41	33.0
Anthracene	0.02 (–0.02, 0.06)	0.14 (0.06, 0.22)	0.30 (0.17, 0.42)	0.12 (0.04, 0.20)	2.7	1.2	3.5	24	52	21.0
Fluoranthene	0.02 (–0.02, 0.06)	0.26 (0.17, 0.35)	0.26 (0.18, 0.35)	0.06 (0.001, 0.12)	1.2	1.4	3.2	43	44	10.0
Pyrene	0.02 (–0.01, 0.04)	0.15 (0.09, 0.21)	0.25 (0.20, 0.31)	0.02 (0.02, 0.03)	1.6	1.3	3.7	34	57	5.1
Benzo[a]anthracene	0.04 (–0.03, 0.11)	0.32 (0.20, 0.44)	0.46 (0.36, 0.56)	0.04 (0.03, 0.06)	1.4	1.3	4.4	37	53	5.2
Chrysene	0.02 (–0.02, 0.07)	0.24 (0.16, 0.32)	0.31 (0.25, 0.37)	0.01 (0.01, 0.02)	1.2	1.4	4.2	41	53	2.1
Benzo[b]fluoranthene	0.03 (–0.03, 0.10)	0.37 (0.26, 0.48)	0.34 (0.27, 0.42)	0.02 (0.02, 0.03)	0.9	1.4	4.3	48	45	3.2
Benzo[k]fluoranthene	0.04 (–0.04, 0.11)	0.39 (0.28, 0.51)	0.36 (0.28, 0.44)	0.03 (0.02, 0.04)	0.9	1.4	4.6	48	44	4.2
Benzo[a]pyrene	0.05 (–0.04, 0.14)	0.39 (0.27, 0.52)	0.42 (0.33, 0.52)	0.05 (0.04, 0.07)	1.1	1.4	5.5	43	46	5.6
Indeno[1,2,3-c,d]pyrene	0.05 (–0.04, 0.14)	0.38 (0.26, 0.50)	0.41 (0.32, 0.49)	0.03 (0.02, 0.04)	1.0	1.4	6.2	43	47	3.5
Dibenzo[a,h]anthracene	0.05 (–0.03, 0.12)	0.29 (0.18, 0.40)	0.43 (0.34, 0.53)	0.04 (0.03, 0.05)	1.4	1.3	5.7	36	54	4.6
Benzo[g,h,i]perylene	0.06 (–0.02, 0.14)	0.26 (0.18, 0.34)	0.29 (0.23, 0.35)	0.02 (0.01, 0.02)	1.0	1.4	9.2	42	46	2.5
PAHs are ordered by molecular weight from lightest to heaviest. aModel 1 was fit for 590 observations including 405 samples collected from 204 homes during repeat sampling rounds, 89 samples from homes that were sampled once, 56 duplicate samples, and 40 interbatch quality control replicates. bλ = (σˆ2WH + σˆ2WS)/(σˆ2BR + σˆ2BH). cORBiased = exp [ln(ORTrue)/(1 + λ)], ORTrue = 2.0.										

For each PAH, when within-household variability was estimated separately for cases and controls (model 2), the within-household variability was larger in case households than in control households (range, 3–75% increase; average, 31% increase; data not shown).

*Mixed-effects modeling*. Table 4 shows estimated variance components for each PAH from the hierarchical random-effects model without covariates (model 1) and the mixed-effects models that included explanatory variables (models 3–6). In model 3, including census tract rankings of ambient air PAH concentration estimates (i.e., neighborhood covariates) explained 14–100% of the regional variability in residential-dust PAH concentrations. In model 4, including smoking habits, residential construction date, residence type, shoe removal habits, carpet coverage, residential square footage, and heating practices (i.e., residential covariates) explained 5–27% of the intraregional between-household variability in residential-dust PAH concentrations. In model 5, including the date of dust collection and the sequence of dust analysis (i.e., temporal covariates) explained –1 to 15% of the within-household variability in residential-dust PAH concentrations over time (where negative values indicate that within-household variability was smaller in model 1 compared with model 5; i.e., the covariates were not informative). In model 6, including each of the neighborhood, residential, and temporal covariates explained 59–100% of the regional variability, 3–28% of the intraregional between-household variability, and –4 to 14% of the within-household variability in residential-dust PAH concentrations over time.

**Table 4:** Changes in estimated variance components from the random-effects modela to the mixed-effects models.

PAH	Model 1: random effects, no covariates			Model 3: mixed effects, neighborhood covariates		Model 4: mixed effects, residential covariates		Model 5: mixed effects, temporal covariates		Model 6: mixed effects, all covariates					
	σ2BR	σ2BH	σ2WH	σ2BR	%BR	σ2BH	%BH	σ2WH	%WH	σ2BR	%BR	σ2BH	%BH	σ2WH	%WH
Phenanthrene	0.01	0.13	0.26	0	100	0.13	5	0.26	–1	0	100	0.13	5	0.27	–3
Anthracene	0.01	0.14	0.32	0.005	68	0.13	5	0.31	3	0.002	86	0.14	3	0.31	2
Fluoranthene	0.03	0.26	0.27	0.02	14	0.20	22	0.27	0	0.01	59	0.20	23	0.27	–3
Pyrene	0.02	0.15	0.26	0.01	63	0.12	17	0.26	2	0.005	75	0.12	16	0.26	0
Benzo[a]anthracene	0.04	0.33	0.47	0.02	52	0.24	25	0.47	–1	0	100	0.24	28	0.48	–4
Chrysene	0.03	0.23	0.31	0.02	33	0.17	27	0.31	–1	0.002	90	0.17	27	0.31	–3
Benzo[b]fluoranthene	0.04	0.34	0.34	0.02	56	0.27	20	0.33	2	0	100	0.26	22	0.34	0
Benzo[k]fluoranthene	0.04	0.37	0.36	0.03	18	0.30	19	0.34	5	0.004	89	0.30	19	0.35	3
Benzo[a]pyrene	0.06	0.37	0.43	0.02	65	0.31	18	0.42	3	0	100	0.29	22	0.43	1
Indeno[1,2,3-c,d]pyrene	0.06	0.35	0.40	0.01	77	0.30	16	0.37	9	0	100	0.29	19	0.37	8
Dibenzo[a,h]anthracene	0.05	0.27	0.42	0.01	86	0.23	16	0.36	15	0	100	0.24	13	0.36	14
Benzo[g,h,i]perylene	0.06	0.24	0.29	0.001	98	0.21	12	0.27	6	0	100	0.19	22	0.27	6
Abbreviations: σ2BR, between-region variance; σ2BH, intraregional between-household variance; σ2WH, within-household variance over time; %BR, percent of between-region variance from the random-effects model (model 1) explained by the neighborhood covariates (i.e., ambient PAH from area and mobile sources) included in the mixed-effects model (model 3 or 6); %BH, percent of intraregional between-household variance from the random-effects model (model 1) explained by the residential covariates (i.e., reported smoking, residence construction date, apartment is residence, shoe removal, carpet coverage, residential square footage, and combustion-based heating) included in the mixed-effects model (model 4 or 6); %WH, percent of within-household variance over time from the random-effects model (model 1) explained by the temporal covariates (i.e., interview and laboratory analysis dates) included in the mixed-effects model (model 5 or 6). PAHs are ordered by molecular weight from lightest to heaviest. aModel 1 and models 3–6 were fit for 451 observations including 405 samples collected from 204 homes during repeat sampling rounds and 46 duplicate samples, and excluding 139 observations without covariate data (40 interbatch quality control replicates and 89 samples with 10 duplicates from homes that were sampled during round 1 only).															

Table 5 shows the percent change in concentrations of each PAH associated with a unit increase in each of the fixed effects included in the saturated hierarchical mixed-effects model (model 6). Participants who reported cigarette smoking, living in an older home, or living in an apartment generally had higher PAH concentrations in their residential dust. Ambient air PAH concentrations from area source emissions and, to a lesser extent, ambient air PAH concentrations from mobile source emissions, were associated with higher concentrations of some PAHs in residential dust. On average, households in the metropolitan San Francisco Bay area had higher PAH concentrations in their dust than households elsewhere in our study population (for example, see results for benzo[*g,h,i*]perylene in Figure 1). This region was also the most densely populated, with the most PAHs emitted from area and mobile sources, and the oldest homes (data not shown). The relationship between ambient air PAH concentrations and residential-dust PAH concentrations was strongest for benzo[*g,h,i*]perylene (*p* < 0.01). There was evidence of a decreasing trend in residential-dust PAH concentrations over time for the heavier PAHs (benzo[*b*]fluoranthene, benzo[*k*]fluoranthene, benzo[*a*]pyrene, indeno[*1,2,3-c,d*]pyrene, dibenzo[*a,h*]anthracene, and benzo[*g,h,i*]perylene).

**Table 5:** Percent change*^a^* (*p*-values) in dust concentrations of PAHs associated with a unit increase in each of the fixed effects included in the saturated hierarchical mixed-effects model (model 6*^b^*).

PAH	Neighborhood covariates		Residential covariates							Temporal covariates	
	Ambient PAH from area sources, per ΔIQRc	Ambient PAH from mobile sources, per ΔIQRd	Reported smoking	Residence construction date, per 10 years	Apartment residence	Shoe removal	Carpet coverage < 25%	Residential area < 1,750 ft2	Combustion-based heating	Interview date, per year	Laboratory analysis date, per 30 batches
Phenanthrene	3 (0.84)	7 (0.54)	27 (0.04)*	–2 (0.35)	22 (0.38)	–4 (0.67)	8 (0.47)	4 (0.67)	3 (0.77)	–1 (0.59)	–4 (0.60)
Anthracene	8 (0.51)	16 (0.15)	39 (0.005)*	2 (0.37)	45 (0.08)	4 (0.67)	7 (0.52)	0 (0.99)	–3 (0.76)	1 (0.23)	–13 (0.07)
Fluoranthene	12 (0.32)	–7 (0.53)	47 (0.001)*	–6 (0.01)*	57 (0.04)*	–5 (0.53)	17 (0.13)	1 (0.93)	7 (0.43)	0 (0.95)	–11 (0.10)
Pyrene	11 (0.31)	5 (0.63)	33 (0.005)*	–3 (0.07)	52 (0.02)*	–4 (0.59)	10 (0.29)	–2 (0.79)	4 (0.63)	1 (0.19)	–11 (0.07)
Benzo[a]anthracene	25 (0.10)	4 (0.74)	59 (0.001)*	–5 (0.04)*	103 (0.01)*	–10 (0.26)	22 (0.10)	0 (0.97)	11 (0.28)	–1 (0.39)	–6 (0.50)
Chrysene	16 (0.18)	–1 (0.94)	44 (0.001)*	–6 (0.01)*	73 (0.01)*	–9 (0.24)	21 (0.06)	–3 (0.70)	10 (0.22)	1 (0.62)	–4 (0.52)
Benzo[b]fluoranthene	31 (0.04)*	2 (0.84)	50 (0.002)*	–5 (0.03)*	80 (0.01)*	–10 (0.21)	14 (0.26)	1 (0.91)	9 (0.33)	–3 (0.01)*	–2 (0.81)
Benzo[k]fluoranthene	30 (0.05)	–5 (0.72)	49 (0.004)*	–5 (0.03)*	90 (0.01)*	–10 (0.23)	16 (0.22)	2 (0.83)	10 (0.29)	–3 (0.01)*	14 (0.08)
Benzo[a]pyrene	39 (0.02)*	11 (0.44)	47 (0.01)*	–5 (0.07)	122 (0.002)*	–8 (0.37)	11 (0.40)	–3 (0.78)	12 (0.26)	–3 (0.01)*	3 (0.76)
Indeno[1,2,3-c,d] pyrene	42 (0.01)*	16 (0.24)	48 (0.004)*	–4 (0.08)	100 (0.01)*	–12 (0.15)	10 (0.43)	–5 (0.62)	10 (0.28)	–5 (< 0.001)*	4 (0.61)
Dibenzo[a,h]anthracene	31 (0.03)*	20 (0.13)	47 (0.003)*	–4 (0.08)	77 (0.02)*	–13 (0.11)	10 (0.41)	–3 (0.74)	12 (0.21)	–4 (< 0.001)*	29 (0.001)*
Benzo[g,h,i]perylene	37 (0.004)*	37 (0.002)*	29 (0.02)*	–3 (0.17)	77 (0.01)*	–8 (0.23)	3 (0.73)	–9 (0.26)	10 (0.23)	–4 (< 0.001)*	–8 (0.19)
IQR, interquartile range. PAHs are ordered by molecular weight from lightest to heaviest. aPercent change = 100 × [exp(β)–1], where β are the regression coefficients from model 6 (i.e., r1’-r11’). bModel 6 was fit for 451 observations including 405 samples collected from 204 homes during repeat sampling rounds and 46 duplicate samples, excluding 139 observations without covariate data (40 interbatch quality control replicates and 89 samples with 10 duplicates from homes that were sampled during round 1 only). cPercent change in dust concentrations of PAHs associated with an IQR increase in ambient PAH from area sources [i.e., from 25th percentile (0.73 ng/m3) to 75th percentile (2.5 ng/m3)]. dPercent change in dust concentrations of PAHs associated with an IQR increase in ambient PAH from mobile sources [i.e., from 25th percentile (2.2 ng/m3) to 75th percentile (9.2 ng/m3)]. *p < 0.05.											

When model 6 was fit for cases and controls separately, many of the fixed effects produced were similar to those shown in Table 5 (data not shown). However, the decreasing time trend in the higher molecular weight PAHs was more apparent in the case households (2–3 times steeper decline in case households). Moreover, inverse relationships between PAH concentrations and residence construction date as well as residence square footage were significant (*p* < 0.05) only in case households (for seven and three PAHs, respectively) (data not shown).

Model 7 showed that decreases in PAH concentrations over time were more evident in the San Francisco Bay area and the Sacramento Valley (data not shown). For example, concentrations of benzo[*g,h,i*]perylene decreased in the metropolitan San Francisco Bay area, the northern San Francisco Bay area, and the Sacramento Valley (changes of –6%, –5%, and –5% per year, respectively), but not in the San Joaquin Valley, the California Central Coast, or the Sierra Mountains (changes of –1%, 1%, and 3% per year, respectively).

For 11 of the 12 PAHs analyzed using model 8, after adjustment for neighborhood, residential, and temporal covariates, there was greater within-household variability in dust samples collected from households at intervals of ≥ 6 years than there was in dust samples collected from households at intervals of < 6 years [see Supplemental Material, Table S1 (http://dx.doi.org/10.1289/ehp.1205821)]. Moreover, in 7 of the 12 PAHs analyzed, the within-household variability increased with the interval between repeat sample collections. For example, the within-household variance components for benzo[*a*]pyrene were 0.39, 0.46, and 0.56 for households sampled at intervals of < 4 years, 4–6 years, and ≥ 6 years, respectively.

## Discussion

To use residential-dust measurements to assess exposures, we must first characterize the reliability of these measures. We observed substantial variability in concentrations of PAHs measured in replicate quality control samples. To some extent, the analytical variability observed in our study is attributable to the heterogeneous nature of residential dust and the resultant heterogeneous distribution of chemicals in residential-dust samples. Although our dust preparation protocol used a mechanical sieve shaker to homogenize household vacuum cleaner dust, the SRM 2585 dust preparation protocol included additional homogenization using a modified food processor, a compressed air jet, and a cone blender. Additional dust homogenization appeared to improve analytical reproducibility, and we recommend that future investigators homogenize each residential-dust sample using a commercial stainless steel blender.

The observed interbatch variability in levels of low-molecular-weight PAHs measured in replicate quality control dust samples may have resulted from the sporadic contamination of samples via laboratory air. Alternatively, the large interbatch variability may have resulted from the sporadic loss of these more volatile PAHs during the evaporation steps of the analytical protocol. Future investigators should use a labeled analog of these lower-molecular-weight PAHs as an internal standard to adjust for analyte losses during evaporation and to reduce analytical variability.

Together, analytical variability and within-household variability over time were at least 90% as large as the between-household variability for each PAH. When estimating health effects related to a chemical exposure, large variance ratios translate to imprecise exposure classification, which tends to result in the underestimation of relative risks (Armstrong 1998). For example, we observed that the variance in phenanthrene concentrations measured in repeat dust samples collected from the same household over a period of several years was three times as great as the variance in mean phenanthrene concentrations from different households across the study population. For exposures estimated using a single dust sample, a variance ratio of λ = 3 would be expected to result in an estimated odds ratio (OR) of 1.2 given a true OR of 2.0. In contrast, for higher molecular weight PAHs including benzo[*b*]fluoranthene, benzo[*k*]fluoranthene, benzo[*a*]pyrene, indeno[*1,2,3-c,d*]pyrene, and benzo[*g,h,i*]perylene, we would expect the variance in PAH concentrations measured in repeat dust samples collected from the same household over several years to be roughly equal to the variance in mean PAH concentrations in different households across the study population and the attenuation of risk estimates would be expected to be less extreme.

If past levels of chemical exposures are of interest and sample collection must be carried out after disease diagnosis (e.g., case–control studies), large unexplained within-household variability over time is problematic. We found that within-household variability was greatest in dust samples collected from households at intervals of ≥ 6 years (even after adjusting for PAH time trends). We suggest that investigators who plan to use residential dust to estimate past levels of PAH contamination in retrospective studies should start sampling as soon as possible after participant enrollment and should complete sampling no more than 5 years after the time period of interest.

We found that case households had more within-household variability in PAH concentrations in the years after disease diagnosis than control households. Moreover, we found that PAH concentrations in case households decreased faster in the years after disease diagnosis than in control households. Our findings highlight the importance of collecting samples as soon as possible after case diagnosis in case–control studies to minimize the impact of changes in case (or case parents’) behavior.

If long-term average chemical exposures are of interest and prospective sample collection is feasible (e.g., cohort studies), investigators can improve the precision of their exposure estimates and limit the attenuation of observed risk estimates by making repeated exposure measurements on each study participant. Because analytical variability was relatively small compared with the variability within households over time, this strategy of analyzing repeat dust samples would increase precision more efficiently than analyzing several replicates for each dust sample.

Median concentrations of PAHs measured in residential dust from homes in our study were similar to levels measured in other California homes (Hoh et al. 2012; Whitehead et al. 2012). However, residential-dust PAH levels in our study were lower than those reported in most other states (Whitehead et al. 2011) and may reflect relatively low prevalence of cigarette smoking in California (Centers for Disease Control and Prevention 2009) as well as limited use of coal tar in California for sealing asphalt surfaces (Van Metre et al. 2009).

Using a pooled analysis, previous investigators have suggested that urban locations have higher concentrations of PAHs in residential dust (Maertens et al. 2004). Likewise, we found that neighborhoods with more PAH emissions from area sources (i.e., dense urban neighborhoods with many residential and commercial buildings) had higher concentrations of most PAHs in their dust, and that emissions from mobile sources (i.e., traffic) also contributed to the level of some PAHs in residential dust. These neighborhood-level covariates explained a large portion of the regional variability in residential-dust levels of PAHs. Because urban density and traffic density were highly correlated (*r*_S_ = 0.72), our mixed-effects models may not have resolved the distinct contribution of area and mobile emissions to PAH contamination in the residential environment. Indeed, it is likely that both traffic and diffuse urban emissions are important sources of PAH in residential dust. The influence of outdoor PAH sources appeared to be greatest for the higher molecular weight PAHs, consistent with earlier findings (Naumova et al. 2002).

Several residential characteristics explained some of the variability in residential-dust PAH levels between households within the same region. Hoh et al. (2012) reported elevated PAH concentrations in residential dust from California homes occupied by smokers. Likewise, we found that residents who reported regular cigarette smoking at their residence (indoor or outdoor) had significantly higher PAH concentrations in their dust than their nonsmoking counterparts. Interestingly, most regular smokers reported only smoking outdoors (4 households reported indoor and outdoor smoking, and 30 households reported outdoor smoking only), which indicates that outdoor smoking can result in elevated indoor concentrations of PAH. This finding is consistent with those of Matt et al. (2004), who reported that nicotine concentrations in residential dust were elevated in homes of participants who only smoked outdoors.

Van Loy et al. (2001) estimated that semi-volatile organic compounds (e.g., nicotine) that are sorbed onto household surfaces such as carpets and painted wallboards could persist for months in the indoor environment. Similarly, we found evidence suggesting that PAHs can accumulate in carpets and possibly other household surfaces over time because residents living in older homes had significantly higher PAH dust concentrations than those in newer homes. In addition, apartment dwellers had significantly higher PAH concentrations than their counterparts living in single-family homes. The disparity between PAH levels in apartments and single-family homes did not appear to be explained by the residential square footage. Apartments may have a higher potential for PAH contamination due to higher residential density or turnover. High-density residences may concentrate more PAH sources in a relatively small space, whereas high resident turnover could increase the potential for residual PAH contamination from previous occupants. In contrast to our earlier report that gas heating was associated with higher residential-dust PAH concentrations (Whitehead et al. 2009), the presence of two or more forms of combustion-based heating was not predictive of PAH levels.

In large health-effects studies, it may be expensive and time-consuming to analyze PAHs in a dust sample collected from each study home. An appealing alternative would be to use a small number of dust measurements to fit a statistical model that could predict indoor PAH levels using self-reported information and preexisting ambient PAH estimates. Using cross-validation, we found that such a model was only marginally predictive of observed PAH levels (Whitehead et al. 2009). Because the fully saturated model 6 also explained a relatively small amount of the variability between homes, we recommend that PAH concentrations be measured directly in dust samples for use in health-effects studies.

Although concentrations of PAHs in residential dust were moderately correlated between sampling rounds, there was substantial unexplained within-household variability. One factor that explained a small portion of the within-household variability was the date of dust collection. Residential-dust levels of the six higher-molecular-weight PAHs appeared to decrease over time in our study—a trend that was particularly evident in the geographic regions with higher residential-dust PAH concentrations. Across California, the mean ambient air concentrations of these six PAHs decreased substantially (e.g., from 0.74 to 0.13 ng/m^3^ for benzo[*a*]pyrene) from 1989 to 2004 (California Environmental Protection Agency 2013). The concordant decrease in both ambient and residential-dust PAH levels provides further evidence that outdoor PAH sources influence indoor concentrations of PAHs.

In contrast to previous findings (Mukerjee et al. 1997), we did not observe a relationship between PAH levels and the season of dust collection. However, vacuum cleaners from our study generally contained dust that had been collected over the course of at least 1 month (i.e., median time since last vacuum bag replacement was 1 month and maximum time was 1.5 years). Thus, any seasonal trends that may have been present would have been obscured by our sampling method.

Although we collected repeated residential dust samples over long time intervals (up to 8 years) our sampling strategy was limited to a maximum of two samples per household, and we did not collect repeat samples from homes over short intervals of time. Consequently, we were unable to estimate the short-term temporal variability of PAHs in residential dust for this study population (i.e., month-to-month variability). Because of the sampling method, we were unable to investigate the spatial variability of PAH concentrations in dust collected from different rooms in the same house.

In summary, we used repeated residential-dust samples collected at intervals of 3–8 years to characterize the long-term variability of PAH concentrations. Although PAH concentrations were correlated within households between sampling rounds, substantial within-household variability was observed, which was comparable in magnitude to the between-household variability and much larger than the within-sample analytical variability. Major sources of PAH contamination included indoor and outdoor smoking and urban emissions, including traffic. Long-term trends toward lower PAH concentrations were observed across California. Our findings indicate that it may be feasible to use residential dust for retrospective assessment of PAH exposures in studies of health effects, especially if dust samples can be collected within 5 years of the relevant exposure.

## Supplemental Material

(729 KB) PDF
