# Supplementary material for: Polycyclic Aromatic Hydrocarbons in Residential Dust: Sources of Variability
Source: Environ Health Perspect. 2013 Mar 5;121(5):543–50. doi: 10.1289/ehp.1205821 (PMC3672903; doi:10.1289/ehp.1205821)
Supplement: (729 KB) PDF [file ehp.1205821.s001.pdf]

*Environmental Health Perspectives doi:10.1289/ehp.1205821*

# SUPPLEMENTAL MATERIAL

## Polycyclic Aromatic Hydrocarbons in Residential Dust: Sources of Variability

Todd P. Whitehead, Catherine Metayer, Myrto Petreas, Monique Does, Patricia A. Buffler, Stephen M. Rappaport

## TABLE OF CONTENTS

Quality control samples ........ 2
Questions used to create variables for mixed-effects models ........ 2
Random-effects Model 2 ........ 3
Mixed-effects models ........ 3
Supplemental Material, Table S1 ........ 7

*Quality control samples*

To compare the magnitude of variability observed in the 4 types of quality control samples we calculated the relative percent difference (RPD) between matched samples. For duplicate samples, we simply report the average of each RPD for each duplicate pair and each PAH (*i.e.*, for each of 12 PAHs, the average of 56 RPDs calculated for 56 duplicate sample pairs). For the 40 inter-batch quality control replicates and 17 NIST SRM 2585 replicates, in order to calculate an average RPD value, we used a random sampling routine with replacement (*i.e.*, for each PAH, we randomly selected 2 inter-batch quality control replicates, calculated an RPD between the 2 concentrations, replaced the 2 replicates in the sampling pool, repeated the sampling 10,000 times, and averaged the RPDs over all the iterations).

*Questions used to create variables for mixed-effects models*

- Smoking:
    - "*During the time you have lived in this home, have you or anyone else regularly, that is once a week or more, smoked cigarettes, pipes or cigars inside this home?*"
    - "*During the time you have lived in this home, have you or anyone who lives in this home regularly smoked cigarettes, pipes or cigars outside of this home (car, work, yard, deck)?*"
    - Response of "*No*" is the reference category.
- Heating used at home:
    - "*In the last 12 months did you use electric heat to heat your home?*"
    - "*In the last 12 months did you use kerosene heat to heat your home?*"
    - "*In the last 12 months did you use a radiator or steam heat to heat your home?*"
    - "*In the last 12 months did you use gas heat (including a gas fireplace) to heat your home?*"
    - "*In the last 12 months did you use a wood-burning stove to heat your home?*"
    - "*In the last 12 months did you use a wood-burning fireplace to heat your home?*"
    - Responses of "*electric heat*" only or "*electric heat*" plus one other heating source are included in the reference category.
- Shoe removal:
    - "*In the last 12 months, did all of the people who lived in this home usually take off their shoes when entering the home?*"
    - Response of "*No*" is the reference category.
- Residence construction date:
    - "*In what year was your home built?*"
    - Modeled as a continuous variable.
- Residence type:
    - "*Is this residence best described as a single family residence?*"

    - "*Is this residence best described as a duplex/townhouse?*"
    - "*Is this residence best described as an apartment/condominium?*"
    - "*Is this residence best described as a mobile home?*"
    - Responses of "single family residence", "duplex/townhouse", or "mobile home" were included in the reference category.
- Residence square footage:
    - "*About how many square feet is your residence (include all heated areas in the home or apartment that are suitable for year-round use)?*"
    - Response of ≥1750 sf is the reference category.
- Carpet coverage:
    - "*Approximately what percentage of your home has carpet?*"
    - Response of ≥25% is the reference category.

*Random-effects Model 2*

In Model 1 we assume equal within-household variability in case and control homes; however to test this assumption we apportion the observed variance in PAH concentrations into three components describing between-household variability, within-household variability in case households, and within-household variability in control households,

$$Y_{ij} = ln(X_{ij}) = \mu_Y + b_i + E_{cases}e_{ij} + E_{cont}e'_{ij} \qquad [2]$$

for $i$ = 1,2,…,293 households; $j$ = sampling round 1 or 2, where:

$X_{ij}$ = the residential-dust PAH concentration for the $i^{th}$ household on the $j^{th}$ repeated measurement;
$b_i$ = $\mu_{Yi}$ -$\mu_Y$, and represents the random deviation of the $i^{th}$ household's true mean (logged) residential-dust PAH concentration, $\mu_{Yi}$, from $\mu_Y$;
$e_{ij}$ = $Y_{ij}$ - $\mu_{Yi}$, and represents the random deviation of the observed (logged) residential-dust PAH concentration, $Y_{ij}$, from $\mu_{Yi}$ for the $i^{th}$ case household,
$e'_{ij}$ = $Y_{ij}$ - $\mu_{Yi}$, and represents the random deviation of the observed (logged) residential-dust PAH concentration, $Y_{ij}$, from $\mu_{Yi}$ for the $i^{th}$ control household,
$E_{cases}$ = 1 for case households, 0 for control households,
$E_{cont}$ = 1 for control households, 0 for case households.

*Mixed-effects models*
We used mixed-effects models to identify sources of variability for each hierarchical level. In addition to the Model 1 random effects, we included two fixed effects for neighborhood-level covariates in Model 3; namely, the rank order of estimated ambient concentrations of PAH attributable to emissions from area sources ($C_{PAH1}$) and the rank order of estimated ambient concentrations of PAH attributable to emissions from mobile sources ($C_{PAH2}$) for the census tracts in the study,

$$Y_{hijk} = \mu_Y + r_1 C_{PAH1} + r_2 C_{PAH2} + b_h + b_{hi} + b_{hij} + e_{hijk} \quad [3]$$

where:

$C_{PAH1}$ = the rank order of estimated ambient concentrations of PAH attributable to emissions from area sources for the census tracts in the study population based on the EPA National-Scale Air Toxics Assessment;
$C_{PAH2}$ = the rank order of estimated ambient concentrations of PAH attributable to emissions from mobile sources for the census tracts in the study population based on the EPA National-Scale Air Toxics Assessment;
$r_1$ and $r_2$ = regression coefficients for the neighborhood-level covariates.

Likewise, in addition to the Model 1 random effects, we included seven fixed effects for residential covariates in Model 4; namely regular smoking inside or outside of the residence ($C_{Smk}$), residence construction date ($C_{Con}$), residence is apartment or condominium ($C_{Apt}$) , regular shoe removal by residents in home ($C_{Sho}$), less than 25% of residence is carpeted ($C_{Crp}$), residence square footage is less than 1750 sf ($C_{SF}$), and residence has at least two forms of combustion-based heating including gas or kerosene heat, fireplace, wood-burning stove, or steam radiator ($C_{Ht}$),

$$Y_{hijk} = \mu_Y + r_3 C_{Smk} + r_4 C_{Con} + r_5 C_{Apt} + r_6 C_{Sho} + r_7 C_{Crp} + r_8 C_{SF} + r_9 C_{Ht} + b_h + b_{hi} + b_{hij} + e_{hijk} \quad [4]$$

where:

$C_{Smk}$ = 1 if residents reported regular smoking at their residence (inside or outside), 0 if not;
$C_{Con}$ = Construction date of the residence as reported by residents;
$C_{Apt}$ = 1 if residents reported living in an apartment or condominium, 0 if not;
$C_{Sho}$ = 1 if residents reported regular shoe removal upon entering their home, 0 if not;
$C_{Crp}$ = 1 if residents reported having less than 25% of their home carpeted, 0 if not;
$C_{SF}$ = 1 if residential square footage was reported as less than 1750 sf, 0 if not;
$C_{Ht}$ = 1 if residents reported using at least two forms of combustion-based heating (*i.e.*, gas heating, fireplace, wood-burning stove, steam radiator, or kerosene heat), 0 if not (*e.g.*, electric heat only);
$r_3$, $r_4$, $r_5$, $r_6$, $r_7$, $r_8$, $r_9$ = regression coefficients for the residential covariates.

Similarly, in addition to the Model 1 random effects, we included two fixed effects for temporal covariates in Model 5; namely the date of dust collection ($C_{DC}$) and the sequence of the laboratory analysis ($C_{AS}$),

$$Y_{hijk} = \mu_Y + r_{10} C_{DC} + r_{11} C_{AS} + b_h + b_{hi} + b_{hij} + e_{hijk} \quad [5]$$

where:

$C_{DC}$ = Date of dust collection;
$C_{AS}$ = Laboratory analysis sequence;
$r_{10}$, $r_{11}$ = regression coefficients for the temporal covariates.

The fully saturated Model 6 contained the random effects from Model 1 as well as neighborhood, residential and temporal covariates from Models 3-5,

$$Y_{hijk} = \mu_Y + r'_1 C_{PAH1} + r'_2 C_{PAH2} + r'_3 C_{Smk} + r'_4 C_{Con} + r'_5 C_{Apt} + r'_6 C_{Sho} + r'_7 C_{Crp} + r'_8 C_{SF} + r'_9 C_{Ht} + r'_{10} C_{DC} + r'_{11} C_{AD} + b_h + b_{hi} + b_{hij} + e_{hijk} \quad [6]$$

where:

$r_1'$, $r_2'$, $r_3'$, $r_4'$, $r_5'$, $r_6'$, $r_7'$, $r_8'$, $r_9'$, $r_{10}'$, $r_{11}'$= regression coefficients for the saturated model.

We fit each of the above mixed-effects models (Models 3-6) for 451 observations with covariate data (*i.e.*, 405 samples collected from 204 homes during repeat sampling rounds and 46 duplicate samples) and excluded the 139 observations without covariate data (*i.e.*, 40 inter-batch quality control replicates and 89 samples with 10 duplicates collected during Round 1). For comparison, we re-ran the random-effects model (Model 1) using this set of 451 observations.

In addition to the Model 6 random and fixed effects, Model 7 includes a unique fixed effect for the time trend in PAH for each region,

$$Y_{hijk} = \mu_Y + r''_1 C_{PAH1} + r''_2 C_{PAH2} + r''_3 C_{Smk} + r''_4 C_{Con} + r''_5 C_{Apt} + r''_6 C_{Sho} + r''_7 C_{Crp} + r''_8 C_{SF} + r''_9 C_{Ht} + r''_{11} C_{AD} + r_{12} C_{MBA} C_{TI} + r_{13} C_{NB} C_{TI} + r_{14} C_{SJV} C_{TI} + r_{15} C_{SV} C_{TI} + r_{16} C_S C_{TI} + r_{17} C_{CC} C_{TI} + b_h + b_{hi} + b_{hij} + e_{hijk} \quad [7]$$

where:

$C_{TI}$ = $C_{DC}|j$=2 – $C_{DC}|j$=1 and represents the time interval between sampling rounds;
$C_{MBA}$ = 1 if the residence is in the metropolitan San Francisco Bay area region and 0 if not;
$C_{NB}$ = 1 if the residence is in the northern San Francisco Bay area region and 0 if not;
$C_{SJV}$ = 1 if the residence is in San Joaquin Valley region and 0 if not;
$C_{SV}$ = 1 if the residence is in the Sacramento Valley region and 0 if not;
$C_S$ = 1 if the residence is in the Sierra Mountains region and 0 if not;
$C_{CC}$ = 1 if the residence is in the California central coast region and 0 if not;

$r_1''$, $r_2''$, $r_3''$, $r_4''$, $r_5''$, $r_6''$, $r_7''$, $r_8''$, $r_9''$, $r_{11}''$, $r_{12}$, $r_{13}$, $r_{14}$, $r_{15}$, $r_{16}$, $r_{17}$ = regression coefficients for Model 7.

Finally, in the fully saturated Model 8 we included each of the fixed effects used in Model 6. However, we apportioned the observed variance in PAH concentrations into four components describing between-household variability (in all homes) and within-household variability in three distinct groups of households based on the interval between repeat dust collections,

$$Y_{ij} = \mu_Y + r'''_1 C_{PAH1} + r'''_2 C_{PAH2} + r'''_3 C_{Smk} + r'''_4 C_{Con} + r'''_5 C_{Apt} + r'''_6 C_{Sho} + r'''_7 C_{Crp} + r'''_8 C_{SF} + r'''_9 C_{Ht} + r'''_{10} C_{DC} + r'''_{11} C_{AD} + b_i + E_{Shrt} e'_{ij} + E_{Mid} e''_{ij} + E_{Lng} e'''_{ij} \quad [8]$$

where:

$e_{ij}$, $e'_{ij}$, $e''_{ij}$ = $Y_{ij}$ - $\mu_{Yi}$, and represents the random deviation of the observed (logged) residential-dust PAH concentration, $Y_{ij}$, from $\mu_{Yi}$

$E_{Shrt}$ = 1 if the interval between sample collections was <4 years and 0 if not;
$E_{Mid}$ = 1 if the interval between sample collections was 4-6 years and 0 if not;
$E_{Lng}$ = 1 if the interval between sample collections was ≥6 years and 0 if not;
$r_1''', r_2''', r_3''', r_4''', r_5''', r_6''', r_7''', r_8''', r_9''', r_{10}''', r_{11}'''$ = regression coefficients for Model 8.

**Supplemental Material, Table S1**. Estimated variance components (95% confidence intervals) from Model 8. PAHs are ordered by molecular weight from lightest to heaviest.

| PAH | Between-household Variance | Within-household Variance, Short Sample Collection Interval[a] | Within-household Variance, Intermediate Sample Collection Interval[b] | Within-household Variance, Long Sample Collection Interval[c] |
|---|---|---|---|---|
| Phenanthrene | 0.11 (0.02, 0.21) | 0.39 (0.27, 0.50) | 0.33 (0.22, 0.45) | 0.56 (0.34, 0.78) |
| Anthracene | 0.13 (0.05, 0.21) | 0.38 (0.27, 0.49) | 0.31 (0.20, 0.42) | 0.49 (0.29, 0.68) |
| Fluoranthene | 0.19 (0.10, 0.27) | 0.26 (0.17, 0.35) | 0.35 (0.23, 0.47) | 0.44 (0.28, 0.60) |
| Pyrene | 0.12 (0.06, 0.18) | 0.20 (0.14, 0.26) | 0.26 (0.18, 0.35) | 0.38 (0.25, 0.51) |
| Benzo(*a*)anthracene | 0.24 (0.13, 0.36) | 0.46 (0.32, 0.60) | 0.42 (0.28, 0.56) | 0.69 (0.46, 0.91) |
| Chrysene | 0.17 (0.10, 0.25) | 0.31 (0.21, 0.40) | 0.27 (0.18, 0.35) | 0.42 (0.28, 0.57) |
| Benzo(*b*)fluoranthene | 0.26 (0.16, 0.36) | 0.31 (0.21, 0.41) | 0.36 (0.25, 0.47) | 0.42 (0.27, 0.58) |
| Benzo(*k*)fluoranthene | 0.30 (0.19, 0.41) | 0.31 (0.21, 0.41) | 0.38 (0.26, 0.50) | 0.46 (0.28, 0.63) |
| Benzo(*a*)pyrene | 0.30 (0.18, 0.42) | 0.39 (0.27, 0.52) | 0.46 (0.31, 0.60) | 0.56 (0.37, 0.76) |
| Indeno(*1,2,3-c,d*)pyrene | 0.29 (0.19, 0.40) | 0.35 (0.24, 0.47) | 0.41 (0.28, 0.54) | 0.42 (0.27, 0.58) |
| Dibenzo(*a,h*)anthracene | 0.24 (0.14, 0.33) | 0.33 (0.23, 0.43) | 0.42 (0.29, 0.55) | 0.46 (0.29, 0.63) |
| Benzo(*g,h,i*)perylene | 0.20 (0.12, 0.27) | 0.25 (0.17, 0.33) | 0.29 (0.20, 0.39) | 0.29 (0.19, 0.39) |

[a] Repeat samples were collected <4 years apart
[b] Repeat samples were collected 4-6 years apart
[c] Repeat samples were collected ≥6 years apart
